# Liquid Biopsy in Advanced Prostate Cancer

**DOI:** 10.3390/cancers18091366

**Published:** 2026-04-24

**Authors:** Pilar Mediavilla-Medel, Natalia García-Simón, Aránzazu González-del-Alba, Atocha Romero

**Affiliations:** 1 Liquid Biopsy Laboratory, Department of Medical Oncology, Hospital Universitario Puerta de Hierro Majadahonda, Instituto de Investigación Sanitaria Puerta de Hierro-Segovia de Arana, 28222 Majadahonda, Spain; pmediavilla@idiphim.org (P.M.-M.); ngarcia@idiphim.org (N.G.-S.); 2 Department of Medical Oncology, Hospital Universitario Puerta de Hierro Majadahonda, Instituto de Investigación Sanitaria Puerta de Hierro-Segovia de Arana, 28222 Majadahonda, Spain; aranglezalba@yahoo.es

**Keywords:** prostate cancer, liquid biopsy, cell-free DNA, circulating tumor DNA, circulating tumor cells, extracellular vesicles, BRCA, biomarkers

## Abstract

Liquid biopsy has emerged as a transformative tool in oncology, enabling minimally invasive and dynamic characterization of tumor biology. In prostate cancer, marked by high heterogeneity and frequent bone metastases, tissue biopsy is often challenging, highlighting the clinical value of circulating biomarkers. Circulating tumor DNA (ctDNA) is the most clinically advanced analyte, supporting detection of actionable alterations such as BRCA1/2 and ATM mutations, guiding targeted therapies, and enabling real-time monitoring of treatment response and resistance. Circulating tumor cells (CTCs) and extracellular vesicles (EVs) provide complementary insights into tumor biology and disease progression. However, challenges remain, including limited sensitivity in low tumor burden and biological confounders such as clonal hematopoiesis (CH), which can lead to false-positive findings. Emerging approaches, including fragmentomics and methylation profiling, offer improved tumor specificity and may help overcome these limitations. Together, these advances support the integration of liquid biopsy into clinical practice for personalized management and longitudinal monitoring in prostate cancer.

## Simple Summary

Liquid biopsy is transforming oncology by enabling non-invasive analysis of tumor-derived material in blood and other body fluids. In prostate cancer, it complements tissue biopsy by capturing tumor heterogeneity, monitoring disease over time, and detecting actionable mutations and resistance mechanisms. Among liquid biopsy analytes, ctDNA is the most clinically advanced, supporting biomarker testing, treatment selection, and prognosis, especially in metastatic disease. Circulating tumor cells (CTCs) and extracellular vesicles (EVs) provide additional prognostic and biological insights, including AR-V7 detection and RNA-based biomarkers. Despite challenges such as false negatives, low tumor fraction, and lack of standardization, liquid biopsy is becoming an important tool in precision prostate cancer care.

## 1. Background

The bloodstream contains a wide variety of analytes released by different tissues of the body, which can provide valuable information regarding the pathophysiological status of tissues, including tumors [1,2]. Traditionally, blood-based biomarkers, such as specific metabolites and proteins, have played a pivotal role in clinical oncology. In this way, serum prostate-specific antigen (sPSA) has long been used for diagnostic, prognostic, and therapeutic monitoring in prostate cancer [3,4].

**Figure 1:**
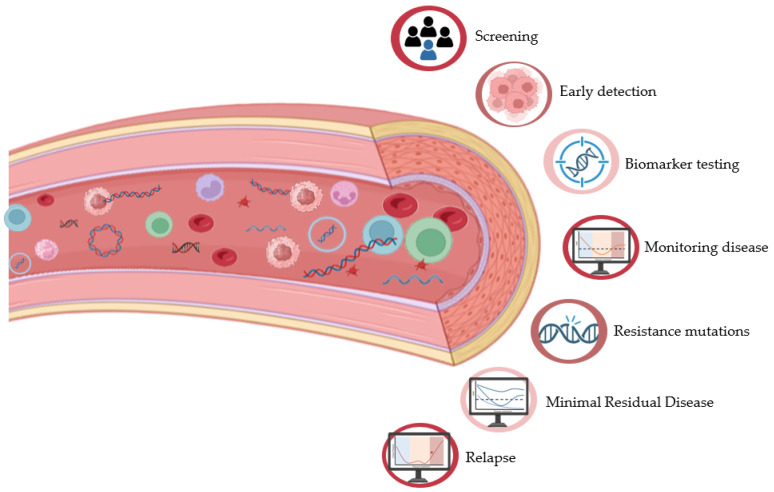
Schematic representation of blood-derived components detectable in liquid biopsy, including cfDNA, RNA fragments, CTCs, EVs, and TEPs and its main applications in clinical and research settings.

More recently, the term liquid biopsy has been defined as a collection of analytical approaches aimed at the detection, characterization, and isolation of tumor-derived material present in biological fluids. While it is predominantly associated with peripheral blood, this concept extends to other biofluids, including urine, cerebrospinal fluid, pleural effusion, and saliva [5,6]. Within the bloodstream, multiple analytes can be interrogated, such as cell-free DNA (cfDNA) and RNA fragments, circulating tumor cells (CTCs), extracellular vesicles (EVs), and tumor-educated platelets (TEPs). These components constitute a heterogeneous and dynamic source of tumor-related information, representing a highly promising platform for non-invasive biomarker discovery and molecular profiling [1,7] (Figure 1).

**Figure 2:**
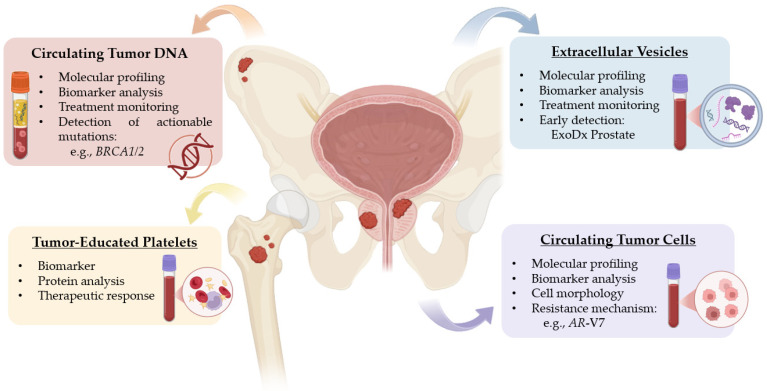
Main clinical applications of liquid biopsy in oncology, including its role in biomarker detection, disease monitoring, and identification of treatment response and resistance mechanisms.

In the present review, we primarily focus on circulating tumor DNA (ctDNA), due to its established role as a diagnostic biomarker, its technical feasibility, and the expanding body of clinical evidence supporting its utility. Additionally, we provide an overview of advances in the analysis of other emerging biomarkers, including CTCs and EVs, which offer complementary information and may enhance the sensitivity and specificity of liquid biopsy approaches (Figure 2).

## 2. Clinical Applications of Liquid Biopsy in Oncology

Tissue biopsy remains the gold standard for molecular and histopathological diagnosis in oncology, as it enables histologic classification, grading, and assessment of non-DNA-based alterations (hormone receptors, protein expression) that liquid biopsy cannot provide [8]. However, it presents several limitations: it is invasive and may not be feasible due to tumor location or patient-related factors. In addition, it often fails to capture the spatial and temporal heterogeneity of tumors [9,10]. Indeed, it has been reported that tissue biopsy yields insufficient material for molecular testing in 20–25% of needle biopsies and is impractical for serial monitoring [11] (Table 1).

In this context, liquid biopsy has emerged as a powerful complementary strategy and a minimally invasive alternative for tumor molecular profiling, enabling the detection of clinically actionable genomic alterations. It also allows for dynamic and longitudinal monitoring of tumor burden and facilitates the identification of resistance-associated mutations during treatment [5,6]. An additional key advantage lies in its ability to capture tumor heterogeneity across multiple metastatic sites. Furthermore, liquid biopsy offers a faster turnaround time compared to tissue-based next-generation sequencing (NGS), with a median of approximately 14 days from blood draw to treatment initiation versus 2–4 weeks for tissue-based approaches [12].

However, liquid biopsy also has important limitations: a negative ctDNA result does not exclude the presence of an actionable mutation; approximately 30% of targetable alterations identified by tissue-based testing are not detected by ctDNA, and around 15% of patients with metastatic cancer may have insufficient ctDNA levels for molecular profiling [13]. Furthermore, sensitivity remains low for copy number variations (CNVs) and gene fusions [14]; (Table 1).

Regarding biomarker testing, the ASCO/CAP Joint Review recommends that ctDNA testing may be used for therapy selection or treatment decisions when tissue is unavailable or insufficient, and repeat biopsy is not feasible; however, it emphasizes that tissue biopsy remains the standard approach for initial pathological diagnosis [15]. In the same way, ESMO guidelines support the use of ctDNA-based liquid biopsy for therapy selection in advanced cancer (e.g., non-squamous NSCLC, prostate cancer, ovarian cancer, and cholangiocarcinoma), when tissue samples are unavailable or inadequate; however, they maintain that tissue-based testing is the preferred standard for most clinical indications [16]. NCCN and ESMO are broadly aligned in supporting the use of ctDNA for therapy selection in advanced disease, while advising against its routine use for minimal residual disease (MRD) assessment, treatment response monitoring, and cancer screening. Both guidelines further emphasize that a negative ctDNA result does not rule out the presence of actionable mutations and should prompt confirmatory tissue-based testing [16,17].

## 3. Importance of Liquid Biopsy in Prostate Cancer

Prostate cancer is a molecularly heterogeneous disease, even at the time of diagnosis. The Cancer Genome Atlas classifies localized prostate cancer into seven molecular subtypes (*ERG*, *ETV1*, *ETV4*, and *FLI1* gene fusions, as well as *SPOP*, *FOXA1*, and *IDH1* mutations), each associated with distinct prognostic features and therapeutic vulnerabilities [18]. Critically, this heterogeneity is present not only between patients but also within individual tumors. A landmark study analyzing more than 630 tumor samples from 52 patients with metastatic prostate cancer demonstrated frequent intra-patient, inter-tumoral molecular subtype heterogeneity, with variable cellular proliferation rates across molecular subtypes and anatomical sites [19]. Of note, more than 80% of prostate tumors are multifocal and frequently arise from distinct clonal lineages [20]. This biological diversity underlies the Gleason grading system, which reflects the architectural and morphological variability across different tumor foci [21].

These foci often exhibit non-overlapping genetic and epigenetic (methylome) profiles, indicating that a single-tissue biopsy may not capture the full spectrum of tumor alterations and can overlook clinically relevant information [22]. This limitation is further compounded by the diffuse and infiltrative growth pattern of prostate cancer, which typically lacks a clearly defined invasive front [20].

This heterogeneity has direct therapeutic consequences. In a study of neoadjuvant ADT plus enzalutamide, tumors with higher baseline histologic and genomic diversity, assessed using the Shannon diversity index and phylogenetic tree reconstruction, had significantly worse pathologic responses with a four-factor predictive model achieving an AUC of 0.89 for poor response [23]. Likewise, a phase III trial has demonstrated that triplet therapy (ADT + ARPI + docetaxel) extends survival beyond 60 months by simultaneously targeting androgen receptor (AR)-dependent cells (through hormonal blockade) and AR-independent/proliferating cells through chemotherapy [18].

Heterogeneity is also a central driver of therapeutic failure in prostate cancer, operating through multiple interconnected mechanisms: pre-existing clonal diversity that harbors treatment-resistant subpopulations, therapy-induced clonal selection and evolution, and lineage plasticity enabling phenotypic switching to AR-independent states [19]. In addition, the 5- and 10-year relative survival rates for local and regional stage are approximately 99% and 95%, respectively, after diagnosis [24]. This prolonged disease trajectory contributes to pronounce even more interpatient genomic and phenotypic variability. Together with the intrapatient heterogeneity described above, this complexity complicates the implementation of personalized treatment strategies [25]. Additionally, it is notable that approximately 70% of prostate cancer metastases develop in bone tissue, a site that poses significant technical and logistical challenges for biopsy acquisition as well as molecular testing, thus limiting comprehensive molecular characterization in many patients [26].

## 4. BRCA Profiling in mCRPC: Implications for Precision Treatment with PARP Inhibitors

Approximately 20–30% of patients with metastatic castration-resistant prostate cancer (mCRPC) harbor pathogenic germline or somatic alterations in genes involved in homologous recombination repair (HRR), with mutations in *BRCA1* and *BRCA2* being the most prevalent, found in approximately in 8–13% of cases [27,28,29,30,31,32]. Multiple clinical trials have demonstrated that this subset of patients derives significant benefit from treatment with poly(ADP-ribose) polymerase inhibitors (PARPi), via a mechanism of synthetic lethality that targets impaired DNA repair pathways [33]. Notably, patients with *BRCA1/2* or *ATM* mutations exhibited a 66% reduction in the risk of disease progression or death compared to those without such alterations [33].

The PROfound clinical trial, along with earlier studies such as TOPARP-A, TOPARP-B and supporting studies such as PROpel, has established the efficacy of olaparib in patients with HRR gene mutations [27,28,33,34]. Based on these findings, the European Medicines Agency (EMA) has approved olaparib for the treatment of patients with mCRPC harboring *BRCA1* or *BRCA2* mutations identified as single nucleotide variations (SNVs) or small insertions/deletions (INDELs). However, this approval does not currently extend to CNVs, due to technical limitations of NGS when applied to formalin-fixed paraffin-embedded (FFPE) tissue samples. DNA derived from FFPE material is often degraded and fragmented, which hampers the accurate detection of CNVs [35]. Alongside olaparib, three additional PARPi namely, rucaparib [29], talazoparib [36], and niraparib [37], have been evaluated and approved in prostate cancer, each characterized by distinct regulatory indications and biomarker criteria (Table 2).

Resistance to PARPi in prostate cancer operates through multiple convergent mechanisms such as restoration of homologous recombination (HR), replication fork stabilization, PARP1 alterations, and cell cycle checkpoint rewiring [38]. Over 40% of *BRCA1/2*-mutated patients develop resistance, and approximately 50% of *BRCA*-mutant patients show upfront (intrinsic) resistance [39]. In the TOPARP-B trial, reversion mutations were identified in 79% of *BRCA2*/*PALB2*-mutated tumors by end of treatment, with the number and timing of reversions significantly associated with both progression-free survival (PFS) and overall survival (OS) (*p* < 0.01) [40]. Similarly, in the TRITON2 trial, *BRCA* reversion mutations were detected in 39/100 patients after progression on rucaparib [41].

A second major resistance axis operates independently of HR restoration. *BRCA1/2*-deficient cells are sensitive to PARPi partly because *BRCA* proteins protect stalled replication forks from nucleolytic degradation by MRE11/EXO1. Resistance arises when fork protection is restored through alternative mechanisms [42]. Loss of pre-replication complex (pre-RC) components (CDT1, CDC6, DBF4) also drives PARPi resistance by promoting rapid resolution of olaparib-induced DNA damage and protecting replication forks from degradation. Notably, approximately 50% of castration-resistant prostate cancer (CRPC) tumors exhibit copy number loss of pre-RC genes, particularly *CDT1*, which may account for the high rate of intrinsic resistance. A pharmacological inhibitor targeting the CDT1/geminin complex (AF615) was able to restore sensitivity to PARPi [39]. More drug–target alterations and alternative repair pathways are explained in Table 3.

## 5. ctDNA for Biomarker Testing in Prostate Cancer

ctDNA is the most extensively studied and clinically validated component of liquid biopsy. Unlike tissue biopsies, ctDNA analysis is not constrained by spatial tumor heterogeneity and can reflect the aggregate genomic landscape of both primary and metastatic lesions, capturing tumor-derived material from multiple anatomical sites [46]. It enables a wide range of applications, including early cancer detection, comprehensive molecular profiling, identification of genomic alterations associated with therapeutic response and resistance, real-time monitoring of tumor dynamics, and assessment of MRD [47,48,49]. Moreover, ctDNA levels correlate strongly with tumor stage and burden, supporting its role as a prognostic biomarker [6].

Over the past 15 years, a broad number of analytical approaches for ctDNA analysis has been developed. Highly sensitive techniques such as droplet digital PCR and BEAMing can detect variant allele frequencies (VAF) as low as ~0.01–0.1%, although they are limited to predefined hotspot mutations [50]. In contrast, NGS-based approaches, including whole-genome sequencing (WGS) and whole-exome sequencing (WES), enable genome- or exome-wide detection of somatic mutations, structural variants, and CNVs. These methods provide more comprehensive tumor profiling but typically require higher ctDNA input [51].

High-sensitivity NGS platforms can detect somatic point mutations and small INDELs at low VAF [52], achieving sensitivities of approximately 91–92% when tracking mutations identified in metastatic prostate tissue in plasma, particularly in patients with higher disease burden or PSA levels above 10 ng/mL [53]. Sensitivity is highest in mCRPC, reflecting increased ctDNA abundance [53]. Detection of CNVs, such as AR amplification, is generally less sensitive than detection of point mutations or INDELs but remains clinically informative. In this regard, targeted NGS identified AR amplification in approximately 31% of mCRPC cases, with sensitivity dependent on TF and sequencing depth [54,55].

Concordance between metastatic tissue biopsy and liquid biopsy (ctDNA analysis) in prostate cancer is generally high, typically ranging from 80% to over 90% for key driver mutations and clinically actionable alterations, provided that the TF is sufficient [9,56]. The sensitivity of ctDNA for detecting *BRCA1/2* and *ATM* mutations is particularly high for nonsense, frameshift, and splice-site variants. In this way, Wyatt et al. reported that 93.6% of mutations identified in metastatic tissue biopsies were also detectable in matched plasma samples [9], while Chi et al. observed detection of 81% of previously characterized mutations in paired tissue specimens, especially in *BRCA1, BRCA2*, and *ATM* genes [57]. In this context, liquid biopsy demonstrated a negative predictive value exceeding 90%, supporting its clinical utility as a non-invasive approach for genomic profiling [58]. Similarly, Goodall et al. [59] showed that both somatic and germline *BRCA2* mutations detected in tumor tissue can be reliably identified in cfDNA, and that patients harboring these alterations derive clinical benefit from PARPi treatment.

In the PROpel study, comprehensive molecular profiling, including *BRCA* testing, was successfully achieved in 93% of patients with negative or inconclusive tissue results using liquid biopsy [34]. Likewise, in the PROfound trial, while only 69% of tissue samples yielded valid NGS results, ctDNA analysis was successful in 100% of cases, highlighting the superior technical feasibility and reproducibility of liquid biopsy in this setting [33].

Regarding CNVs, prostate cancer is characterized by a high burden of such alterations, making CNV profiling particularly informative. In a large ctDNA profiling study (*n* = 3334 mCRPC patients), the most frequent CNVs included *AR* amplification, *MYC* gain, *BRAF* amplification, *PTEN* loss, and *PIK3CA* gain [56]. Notably, when TF is adequate (>2%), copy number profiles show high concordance between matched liquid and solid biopsies, with 88.9% agreement for individual copy number calls in clinically actionable genes [9].

Collectively, liquid biopsy represents a complementary, and in some cases alternative, strategy for molecular profiling in mCRPC. Current clinical guidelines emphasize the importance of dynamic molecular assessment. The ASCO 2026 Living Guideline recommends HRR testing prior to systemic therapy and stratifies PARPi use according to specific gene alterations [60]. Similarly, NCCN guidelines highlight that tumor molecular profiles evolve over time, supporting reassessment at disease progression. To address tumor heterogeneity, a multimodal approach is recommended, incorporating metastatic tissue biopsy as the standard when feasible, ctDNA analysis when biopsy is not possible, and Prostate-Specific Membrane Antigen-PET (PSMA-PET) imaging to evaluate spatial heterogeneity across metastatic sites [17]. In Table 4, there is a summary of the different ctDNA assays available for prostate cancer.

Emerging ctDNA-based omics approaches, including fragmentomics and methylation profiling, offer additional layers of biological information. Fragmentomics leverages cfDNA fragmentation patterns to improve cancer detection and classification. Machine learning models applied to fragmentomics data have shown high accuracy in distinguishing prostate cancer from other malignancies and in identifying specific subtypes, such as neuroendocrine prostate cancer, with area under the curve (AUC) values exceeding 0.98 in validation cohorts [61,62]. Integration with PSA measurements further enhances diagnostic performance for early detection [63]. Additionally, changes in cfDNA concentration and fragmentation profiles enable non-invasive monitoring of disease progression and treatment response [64,65].

Genome-wide methylation profiling in prostate cancer reveals the coexistence of hypermethylation and hypomethylation events [66]. Promoter hypermethylation is associated with gene silencing, whereas global hypomethylation, particularly across large chromatin domains, can contribute to immune evasion by affecting immune-related gene regulation [67]. In metastatic disease, cfDNA exhibits widespread hypermethylation, including at polycomb repressor complex targets, alongside hypomethylation in pericentromeric regions; these patterns correlate with TF and clinical aggressiveness [67].

From a clinical perspective, DNA methylation signatures are increasingly used for risk stratification and prognosis [68]. Importantly, methylation panels can improve diagnostic specificity compared with PSA alone and are detectable in both tissue and cfDNA, enabling non-invasive liquid biopsy approaches for early detection and disease monitoring [68,69].

From a clinical standpoint, beyond its utility for tumor molecular profiling, ctDNA also provides important prognostic information. Although PSA remains a cornerstone in the clinical management of prostate cancer, it lacks cancer specificity, as elevated levels may also occur in benign conditions such as prostatitis and benign prostatic hyperplasia [70]. In contrast, ctDNA offers several advantages, particularly in advanced and mCRPC, as it enables non-invasive, real-time molecular profiling and facilitates the detection of clinically relevant genomic alterations, such as *BRCA1/2* and AR mutations, that can inform targeted therapy selection and reveal mechanisms of resistance [71].

Importantly, Wyatt et al. showed that although ctDNA was detectable in patients with high PSA levels, PSA concentrations did not correlate with ctDNA levels [9]. Elevated baseline ctDNA levels have consistently been associated with poor prognosis in prostate cancer [72,73,74]. Both quantitative and qualitative ctDNA features, including total TF, VAF, and the number of driver mutations, have been independently linked to worse OS and PFS [75,76,77]. Moreover, ctDNA dynamics demonstrate strong prognostic value, correlating with OS and enabling early detection of treatment response or disease progression, often preceding changes in PSA levels [78,79]. In this context, elevated circulating cfDNA levels prior to taxane chemotherapy in patients with mCRPC identify individuals with more aggressive disease, while reductions in cfDNA during the first 3–9 weeks of treatment correlate with therapeutic benefit [80]. Similarly, in the TheraP trial, which compared radioligand therapy (LuPSMA) against cabazitaxel in men with mCRPC progressing after docetaxel, a post hoc ctDNA analysis (*n* = 180, 290 serial samples) showed that low pretreatment ctDNA fraction predicted superior biochemical response (100% vs. 58%; *p* = 0.0067) and PFS (median 14.7 vs. 6.0 months; HR 0.12) on LuPSMA, though this did not extend to OS [81].

### Technical and Pre-Analytical Challenges

Pre-analytical and technical factors critically impact ctDNA testing performance in prostate cancer. Key pre-analytical variables include specimen collection, processing time, storage conditions, patient-related factors, and treatment timing, all of which may reduce sensitivity and lead to false negatives [14]. Plasma is the preferred specimen, requiring prompt separation (within 4–6 h) and appropriate storage to preserve ctDNA integrity. Notably, timing relative to systemic therapy is the most influential modifiable factor, with current guidelines recommending testing at biochemical (PSA) or radiographic progression to maximize yield [17,82].

Other major challenges include low TF and the lack of standardized thresholds. Most platforms require ≥1% TF for reliable mutation detection and >10% for accurate CNV analysis, while low TF significantly increases false-negative rates [83]. Although concordance with tissue is high when TF is adequate (>2%), the use of stringent cutoffs may render up to 50% of samples uninformative and lead to missed low-frequency variants, an issue that can be partially mitigated by ultra-deep sequencing [84].

Another major source of complexity is CH, an age-related process characterized by the expansion of hematopoietic clones carrying somatic mutations [85]. CH-derived variants can be detected in cfDNA and may be misinterpreted as tumor-derived alterations. Indeed, a substantial proportion of mutations identified in cancer patients originate from CH, contributing to analytical noise and potential misclassification [86]. In a large cohort of 17,469 cancer patients, 7608 CH mutations were identified in 26.5% of cases; notably, 14.1% of these variants were also detected in solid tumor sequencing, with nearly half classified as oncogenic and 3.2% linked to targeted therapies, underscoring the risk of erroneous clinical interpretation [87].

Finally, ctDNA analysis may reveal variants unrelated to the underlying malignancy, including incidental findings and germline alterations. The detection of pathogenic variants at high allele frequency is suggestive of germline origin, with important implications for both patient management and familial risk assessment. To guide clinical interpretation, the European Society for Medical Oncology (ESMO) has established recommendations for germline-focused evaluation of tumor-only sequencing data [88]. These guidelines define genes that warrant germline testing irrespective of tumor type, as well as those requiring evaluation only in specific clinical contexts. Notably, *BRCA1* and *BRCA2* are included among the genes recommended for universal germline assessment; therefore, the identification of pathogenic variants in these genes through ctDNA analysis should prompt confirmatory germline testing to inform genetic counseling and clinical decision-making.

## 6. CTCs for Monitoring Prostate Cancer

CTCs are tumor-derived cells that detach from primary or metastatic lesions and enter the bloodstream, offering valuable opportunities for cancer detection, prognostic assessment, and monitoring of therapeutic response [89].

Multiple strategies have been developed for CTC isolation, primarily based on immunoaffinity or physical properties. Immunoaffinity-based approaches include positive enrichment methods such as AdnaTest (Qiagen, Hilden, Germany), MagSweeper (Stanford University, Stanford, CA, USA), and CellSearch (Menarini, Florence, Italy), as well as negative enrichment techniques that deplete non-tumor cells based on size or density differences [90]. Among these, the CellSearch system is the first and only FDA-approved platform for CTC detection and enumeration. It relies on immunomagnetic separation of EpCAM-positive cells, followed by cytokeratin staining and CD45 exclusion to distinguish epithelial tumor cells from leukocytes [89]. In a prospective multicenter study involving 92 metastatic breast cancer patients, this platform demonstrated high reproducibility, with recovery rates of 80–82% and stable CTC counts for up to 72 h under variable storage and transport conditions [91]. Once isolated, CTCs can be analyzed at the genomic, transcriptomic, and proteomic levels, providing insights into tumor biology [92]. Moreover, CTCs provide a unique platform for functional studies, as they can be cultured or used to generate xenograft models, enabling investigation of resistance mechanisms and evaluation of therapeutic strategies [93].

Clinically, CTC enumeration has emerged as a robust prognostic biomarker in mCRPC. CTC counts, assessed as a continuous variable, are strongly associated with clinical outcomes, and early changes following treatment initiation correlate with therapeutic response [94]. In 2014, Goldkorn et al. showed that baseline CTC counts and early increases during docetaxel treatment predicted overall survival [95]. Scher et al. demonstrated that combining CTC enumeration with lactate dehydrogenase (LDH) levels improved survival prediction [96]. Subsequent analyses across five phase III trials confirmed that the absence of CTCs or conversion to ≤4 CTCs was associated with improved overall survival, supporting their role as early response markers [97].

The prognostic value of CTCs has also been observed in metastatic hormone-sensitive prostate cancer (mHSPC), where patients with ≥5 CTCs per 7.5 mL of blood exhibit significantly worse overall survival compared to those with lower counts, improving predictive accuracy beyond standard clinical variables [89]. These findings establish CTC enumeration as a reliable non-invasive prognostic tool.

Finally, in the TOPARP-A and TOPARP-B trials, reductions in CTC counts (≥5 to <5 CTCs per 7.5 mL) were observed in patients who responded to olaparib, and these reductions were associated with improved PFS, OS, and longer durations of response, supporting their utility as a dynamic biomarker for monitoring therapeutic efficacy [27,28].

## 7. Detection of Androgen Receptor Splice Variant 7 (AR-V7) in Prostate Cancer

In prostate cancer, AR-V7 has emerged as a predictive biomarker of resistance to androgen receptor signaling inhibitors (ARSIs). AR-V7 is a constitutively active, ligand-independent isoform of the AR that is rarely expressed in primary, hormone-sensitive prostate cancer but emerges with castration resistance and increases further after exposure to ARSIs such as abiraterone and enzalutamide, particularly in mCRPC [98,99,100,101]. Detection of AR-V7 has been independently associated with lower PSA response rates, shorter PFS, and reduced OS in patients treated with ARSIs [102]. Detection methods for AR-V7 include immunohistochemistry (IHC) using validated monoclonal antibodies, and RNA-based assays such as reverse transcription PCR (RT-PCR) and junction-specific RNA in situ hybridization (RISH) [98,99]. Assays for AR-V7 detection in CTCs have also been developed and applied in clinical studies and specialized clinical settings [98,100].

In a landmark study, Scher et al. validated nuclear-localized AR-V7 expression in CTCs as a predictive biomarker for treatment selection in mCRPC [103]. Their findings demonstrated that patients with AR-V7–positive CTCs derived greater clinical benefit from taxane-based chemotherapy compared to ARSIs such as enzalutamide or abiraterone, supporting the use of AR-V7 status to guide therapeutic decisions.

Along the same lines, the PRESIDE clinical trial assessed the utility of AR-V7 detection in CTCs or plasma to stratify mCRPC patients based on their likely response to continued enzalutamide therapy. Detection of AR-V7 was feasible and allowed for the identification of patients with enzalutamide resistance. Furthermore, a significant correlation was observed between AR-V7–positive CTCs and elevated ctDNA levels, suggesting a possible synergistic role for these biomarkers in molecular stratification [104].

These results are consistent with those reported by Antonarakis et al. in 2017, who were the first to describe AR-V7 detection in CTCs as a negative prognostic biomarker in mCRPC, associated with poor response to ARSIs and worse clinical outcomes [100].

Despite this evidence, AR-V7 is not included as a recommended biomarker in the current NCCN Prostate Cancer Guidelines for routine clinical decision-making, which prioritize clinically actionable somatic alterations in HRR genes, MSI-H/dMMR, and TMB, as these are linked to FDA-approved therapies such as PARPi and pembrolizumab [17].

## 8. Extracellular Vesicles as Emerging Biomarkers in Prostate Cancer Liquid Biopsy

EVs are lipid bilayer-bound nanovesicles, typically ranging from 50 to 1000 nanometers in diameter, that are secreted by all cell types [105]. These vesicles carry a diverse cargo of biomolecules, including proteins, nucleic acids, and metabolites, which they transport to neighboring or distant cells, facilitating intercellular communication. In the context of cancer, tumor-derived EVs play a critical role in promoting tumor progression, immune modulation, and metastasis, acting as messengers that reshape the tumor microenvironment [106].

EVs constitute a highly heterogeneous population, encompassing multiple particles, including exosomes, ectosomes, and microvesicles, which are often classified based on size, origin, and mechanisms of biogenesis [84]. However, distinguishing between EV subtypes remains challenging due to size overlap, shared physical properties, and the lack of definitive molecular markers for specific EV categories [107,108]. Current methods, such as flow cytometry, dynamic light scattering, and nanoparticle tracking, have technical limitations and mainly assess size post-isolation [109].

The clinical use of EVs in prostate cancer is limited by widespread reproducibility issues across the entire workflow (from sample handling and isolation to characterization, cargo analysis, and clinical validation) with no standardized methods currently established for any of these steps [110,111] (Table 5). Furthermore, an important issue across the entire analytical pipeline, is the limited large-scale multicenter validation since most studies are single-center [112].

A universal normalization method for EV experiments has not yet been established, as the optimal approach varies depending on the type of biofluid, sample processing conditions, and the specific target molecule being analyzed. To address these challenges, the Minimal Information for Studies of Extracellular Vesicles (MISEV 2023) guidelines recommend using a combination of physical, biochemical, and functional characterization techniques to improve data reproducibility and transparency [113]. Additionally, emerging consensus highlights the need for robust reference standards and biofluid-specific workflows, particularly in prostate cancer liquid biopsy studies where EV heterogeneity complicates interpretation [114,115].

Tumor-derived EVs contain proteins and microRNAs specific to prostate cancer (such as PCA3, TMPRSS2:ERG, and miR-375), which are useful for diagnosis, prognosis, and treatment monitoring [116,117]. In addition, EVs may have potential utility for detecting fusion transcripts, an approach that has already been demonstrated in other cancer types [118,119].

In prostate cancer, Casanova-Salas et al. analyzed EV-derived DNA (EV-DNA) and RNA (EV-RNA) from plasma of metastatic prostate cancer patients [106]. Despite being ~20 times less abundant than cfDNA, EV-DNA showed strong genomic concordance with tumor tissue (CNA correlation r = 0.87). A TF ≥ 4% in EV-DNA was linked to higher tumor burden (PSA, bone metastases) and worse clinical outcomes, highlighting its promise as a minimally invasive biomarker for disease monitoring [106]. Furthermore, they demonstrated that transcriptome of circulating EVs is enriched in tumor-associated transcripts, captures specific patient and tumor characteristics, and reflects therapy-induced tumor adaptation [106]. These findings led to the development of the ExoDx Prostate (IntelliScore) test, a non-invasive RNA-based EV assay designed to aid decision-making regarding prostate biopsy in men aged 50 years or older with PSA levels between 2 and 10 ng/mL [120]. In a multicenter study involving 1212 patients across 24 urology practices in the United States, the test demonstrated a sensitivity of approximately 90% for detecting high-grade prostate cancer (Gleason score ≥ 7) and a negative predictive value of 89% [121]. While ExoDx Prostate does not replace a biopsy, it serves as a risk stratification tool, helping clinicians determine the necessity of proceeding with biopsy based on the likelihood of clinically significant cancer. Currently, this RNA-based exosome assay is included in the National Comprehensive Cancer Network (NCCN) guidelines for early prostate cancer detection [122].

However, the vast majority of EV biomarkers in prostate cancer remain at the discovery or early verification phase, with only the ExoDx Prostate IntelliScore (EPI) having achieved full clinical validation through multiple prospective multicenter studies and regulatory clearance [121]. The verification phase is where the “reproducibility crisis” has its greatest impact: biomarker candidates identified with one method often fail to replicate when a different method is used, because different techniques yield EVs with different size distributions, purity, and cargo profiles [123].

Several microRNAs associated with prostate cancer, including miR-21-p, miR-375, and miR-141, have been identified in EVs from urine and plasma, showing elevated levels in prostate cancer patients [116,121,124,125]. Several studies have shown that PSA and tetraspanins (CD63, CD9) associated with EVs in urine and plasma are elevated in prostate cancer patients and outperform conventional PSA testing [114,126].

Survivin, a member of the inhibitor of apoptosis protein family, has also been explored as a potential biomarker for prostate cancer. Elevated levels of survivin in plasma-derived EVs, as measured by ELISA, have been observed in prostate cancer patients compared to those with benign prostatic hyperplasia or healthy controls [127]. This result correlates with recent findings by Nanou et al. where a higher amount of tumor-derived EVs were found in the plasma of CRPC patients compared with healthy men, and that an increase in EV numbers was associated with lower OS [128].

AR-V7 mRNA has been detected in plasma and urinary EVs from CRPC patients. Its presence in plasma EVs was associated with shorter PFS and OS, though some studies suggest that CTCs may be more reliable for predicting resistance to AR therapy [129,130,131].

## 9. Conclusions and Future Perspectives

In summary, liquid biopsy is no longer a purely exploratory tool in oncology; it is becoming an essential component of precision cancer care. In prostate cancer, it overcomes major limitations of tissue biopsy by enabling non-invasive, repeatable, and dynamic assessment of tumor burden, molecular evolution, and treatment resistance. Among currently available approaches, ctDNA is the most clinically actionable analyte, already supporting biomarker testing, therapeutic selection, and disease monitoring, while CTCs and EVs add complementary prognostic and biological information that can further refine patient stratification. Although challenges related to sensitivity, standardization, and biological confounders still need to be addressed, the evidence now strongly supports the incorporation of liquid biopsy into routine clinical decision-making.

## Figures and Tables

**Table 1:** Advantages and Limitations of Tissue and Liquid Biopsies.

Tissue Biopsy	Liquid Biopsy
Strengths	
Gold standard Histologic classification Grading Assessment in non-DNA based alterations	Minimally invasive Molecular profiling Actionable genomic alterations Monitoring disease Identification of resistance mutations It captures heterogeneity
Weaknesses	
Invasive Feasibility due to tumor location Patient-related factors frequently insufficient material Unable to capture heterogeneity Frequently inadequate material	False negatives Low tumor fraction (TF) Clonal hematopoiesis Limited sensitivity with CNVs and gene fusions Clinical validation gap

**Table 2:** Main trials of PARPi with or without ARPis.

Clinical Trial	Treatment	FDA-Approved State Indication	Biomarker
PROfound [33]	Olaparib	mCRPC progressed to enzalutamide/abiraterone	*BRCA1/2*, *ATM*, *BARD1*, *BRIP1*, *CDK12*, *CHEK1/2*, *FANCL*, *PALB2*, *RAD51B*, *RAD51C*, *RAD51D*, *RAD54L*
PROpel [34]	Olaparib Abiraterone	mCRPC	*BRCA1/2*
TALAPRO-2 [36]	Talazoparib Enzalitamide	mCRPC	*BRCA1/2*, *ATM*, *ATR*, *CDK12*, *CHEK2*, *FANCA*, *MLH1*, *MRE11A*, *NBN*, *PALB2*, *RAD51C*
MAGNITUDE [37]	Niraparib Abiraterone	mCRPC	*BRCA1/2*
TRITON [29]	Rucaparib	mCRPC treated with AR-therapy and taxane-based chemotherapy	*BRCA1/2*

**Table 3:** Target alteration in prostate cancer and alternatives [43,44,45].

Target Alteration	Alternative Repair Pathway
PARP1 loss or mutation	Eliminates the substrate for PARP trapping, the primary cytotoxic mechanism of PARPi. PARP1 knockout causes cross-resistance to multiple PARPi (olaparib, veliparib, niraparib)
ARH3 loss	Reduces autophagy and confers olaparib resistance; low ARH3 expression is an independent predictor of recurrence in prostate cancer patients.
CHEK2 loss	Confers PARPi resistance by upregulating BRCA2 expression through derepression of the CHEK2-TP53-E2F7 transcriptional axis. Combined PARP + ATR inhibition overcomes this resistance.
G2-M checkpoint override	Olaparib-resistant prostate cancer cells bypass olaparib-induced senescence through G2-M checkpoint override, accumulating and tolerating persistent DNA damage. CDK1 inhibition resensitizes these cells.

**Table 4:** Comparison of liquid biopsy platforms for prostate cancer and multi-cancer applications.

Assay/Platform	Technology	Genes/Scope	LOD (VAF)	FDA Status	Primary Clinical Application	Key Strengths	Key Limitations
FoundationOne Liquid CDx	Hybrid-capture NGS (targeted panel)	324 genes	0.40% (SNVs/indels) 0.37% (rearrangements)	FDA-approved CDx for olaparib (BRCA-mutated mCRPC) and rucaparib	Actionable mutation genotyping HRR status, MSI, bTMB	Broadest FDA-approved gene panel MSI/TMB high reproducibility low false-positive rate	High TF required for CNV; CHIP interference; cost
Guardant360 CDx	Amplicon-based NGS (targeted panel)	55–83 genes	~0.25–0.50%	FDA-approved CDx (pan-tumor)	Actionable mutation genotyping AR monitoring Serial profiling	Rapid turnaround (~7 days) widely available large real-world database (GuardantINFORM)	Smaller panel than F1LCDx; inter-platform concordance with tissue ~40–52%
GuardantOMNI	Hybrid-capture NGS (expanded panel)	500 genes	~0.25%	Research use/clinical trials	Comprehensive genomic profiling in clinical trials	Largest commercial liquid biopsy panel TMB assessment	Higher TMB values vs. tissue (8.6 vs. 3.5 mut/Mb); research-grade
AR-ctDETECT	Targeted cfDNA sequencing (custom panel)	AR + selected cancer genes	Not formally published	Research use	AR resistance monitoring; ctDNA detection prognostication in mCRPC	Specifically designed for prostate cancer AR biology Detects AR-GSRs and ecDNA signatures Validated in phase 3 trial	Not commercially available; research assay
FoundationOne Tracker (Signatera-based)	Tumor-informed mPCR-NGS (16-plex)	16 patient-specific variants from prior tissue CGP	~0.01% (5 MTM/mL)	FDA-approved (pan-tumor MRD) Not prostate-specific CDx	MRD detection Recurrence monitoring Treatment response	Ultra-high sensitivity (0.01%) Specificity 99.6% no need for separate normal tissue sequencing	Requires prior tissue CGP; limited prostate-specific validation data; does not profile new mutations
Signatera Genome	Tumor-informed WGS-based mPCR-NGS (64-plex)	64 patient-specific variants from WGS	≤0.01%	Research/emerging clinical	MRD detection with enhanced sensitivity	4× more variants than exome version 99.8% specificity concordant with Signatera Exome	Requires WGS of tumor+normal; limited prostate-specific data; cost
HERCULES	Amplicon-based targeted sequencing (AmpliSeq HD)	36 prostate cancer-specific genes	0.1% (50% sensitivity) 1% (100% sensitivity)	Research use	Parallel profiling of ctDNA + single CTCs	First panel for simultaneous ctDNA and CTC genomic profiling prostate-specific design	Early validation; small sample sizes; not commercially available
Low-pass/Shallow WGS	Whole-genome sequencing at 0.1–1× depth	Genome-wide CNV profile	N/A (CNV-based)	Research use	Genome-wide CNA burden HRD/TD phenotyping Tumor fraction estimation	Unbiased genome-wide view; cost-effective Detects genomic scars (HRD, TD) No gene-specific bias	Cannot detect SNVs; requires ≥3% TF for standard analysis; bioinformatics-intensive
Deep WGS (30–60×)	Whole-genome sequencing at high depth	All genomic alterations (SNVs, CNVs, SVs, ecDNA)	~0.5–1% (SNVs)	Research use	Comprehensive genomic characterization Clonal architecture Nucleosome footprinting	Most complete genomic view; detects all alteration classes including ecDNA and AR-GSRs enables transcription factor activity inference	Very high cost; requires high TF; long turnaround; not scalable for routine use
Methylation-based: cfMeCaP	Genome-wide methylome profiling (48-region signature)	48 prostate-specific methylation regions	Mutation-independent (methylation-based)	Research use	Prognostication ctDNA detection across disease stages Serial monitoring	95–100% sensitivity in mCRPC Iindependent of somatic mutations Strong prognostic value Works at low TF	Early-stage validation; not yet commercially available
Methylation-based: mDETECT	Multiplexed targeted methylation sequencing (46 PCR probes to 40 regions)	40 prostate-specific methylation regions	Mutation-independent	Research use	Tumor burden tracking PSA-independent monitoring	Works in serum and plasma Resistant to normal DNA contamination Tracks burden in PSA-nonproducing tumors	Small validation cohort; requires further clinical validation
Methylation-based: mm-ddPCR	Multiplex droplet digital PCR (5 methylation biomarkers)	5 CpG regions (ACTRT2, EVX1, HOXD13, DOCK2, HAPLN3)	Single-molecule sensitivity (ddPCR)	Research use	ctDNA detection in mCRPC potential risk stratification	Simple, fast, cost-effective; single-tube assay 95% detection in mCRPC	Limited to 5 targets; no genotyping capability; early validation
Digital PCR (single-gene)	dPCR targeting specific mutations	1–3 targets	0.01–0.1%	Research use	Monitoring known mutations Treatment response tracking	Highest sensitivity for known variants Absolute quantification Low cost per test	Requires prior knowledge of target; no discovery capability; limited multiplexing
Multi-cancer early detection (MCED): Galleri	Targeted methylation (cfDNA bisulfite sequencing)	>100,000 methylation regions; pan-cancer	Mutation-independent	Laboratory Developed Test (CLIA)	Multi-cancer screening	Detects 50+ cancer types with tissue-of-origin prediction High specificity (99.5%)	Low sensitivity for localized prostate cancer; not prostate-specific; cost

**Table 5:** Reproducibility issues and standardization challenges with EVs in prostate cancer.

PRE_Analytical Variables	Isolation Variability	Characterization and Quantification	EV Heterogeneity
Blood collection tube type Storage Time from blood draw to initial centrifugation Urine-specific challenges (hydration, nutrition…) Freeze–thaw cycles	There is no gold standard: - Ultracentrifugation - Density gradient centrifugation - Polymer-based precipitation - Affinity-based capture - Size-exclusion chromatography	Poor reproducibility: - Flow cytometry: most common but incomparable between flow cytometers - Nanoparticle tracking analysis: substantial disagreement between techniques	Exosomes Microvesicles Large oncosomes Affected by clinical variables: stage, treatment

## Data Availability

No new data were created or analyzed in this study. Data sharing is not applicable to this article.

## Abbreviations

The following abbreviations are used in this manuscript:

PSA	Prostate-Specific Antigen
cfDNA	Cell-free DNA
CTCs	Circulating Tumor Cells
EVs	Extracellular Vesicles
TEPs	Tumor-Educated Platelets
ctDNA	Circulating Tumor DNA
CRPC	Castration-Resistant Prostate Cancer
mCRPC	Metastatic Castration-Resistant Prostate Cancer
HRR	Homologous Recombination Repair
PARPi	PARP inhibitors
EMA	European Medicines Agency
SNV	Single Nucleotide Variation
INDEL	insertions/deletions
CNV	Copy Number Variation
NGS	Next-Generation Sequencing
MRD	Minimal Residual Disease
AR	Androgen Receptor
AUC	Area Under the Curve
CH	Clonal Hematopoiesis
ESMO	European Society for Medical Oncology
mHSPC	Metastatic Hormone-Sensitive Prostate Cancer
ARSIs	androgen receptor signaling inhibitors
IHC	Immunohistochemistry
RT-PCR	Real-Time PCR
RISH	RNA in situ hybridization
MISEV	Minimal Information for Studies of Extracellular Vesicles
